# Faster Weight Growth in Invasive Mosquitofish *Gambusia holbrooki* and *Gambusia affinis* (Poeciliidae) Under Climate Change

**DOI:** 10.1002/ece3.72943

**Published:** 2026-01-12

**Authors:** Shanshan Rao, Wenjing Qi, Haochen Cao, Cong Tang, Yujie Xiao, Yuxin Sun, Wen Xiong, Peng Xie, Kun Xu

**Affiliations:** ^1^ Hubei Key Laboratory of Edible Wild Plants Conservation and Utilization, College of Life Sciences Hubei Normal University Huangshi China

**Keywords:** climate change, invasive ability, length‐weight relationship, mosquitofish, native aquatic diversity, Power law

## Abstract

Mosquitofish *Gambusia holbrooki* and *Gambusia affinis* (Poeciliidae) threaten native aquatic diversity globally. Climate change likely increases the body weight and alters the body condition of mosquitofish, resulting in higher invasive ability. The growth of mosquitofish follows the allometric relationship between length (*L*) and weight (*W*), which can be estimated as *W* = *aL* ^*b*^. The values of the scaling exponent *b* among global mosquitofish populations range from 2.68 to 3.76, where *b* > 3 indicates faster growth in weight than in length. The populations with higher values of the scaling exponent b demonstrate stronger body conditions, reproductive ability, and invasiveness. Currently, there is little understanding of how the length‐weight allometries of global mosquitofish populations vary by climate conditions. In this study, we compiled the values of the scaling exponent *b* of 79 mosquitofish populations from six continents and built generalized least squares and random forest regression models on the scaling exponent b with year of sample, elevation, and 11 bioclimatic variables. We find that the populations of *G. affinis* are more sensitive to climatic variation than *G. holbrooki* in terms of length‐weight allometries. Under climate change, the populations of *G. affinis* , especially those in East Asia and Eastern Europe, are expected to grow faster in weight than in length, posing greater threats to native aquatic diversity. This finding informs the need for early identification and eradication of mosquitofish in newly invaded aquatic ecosystems under climate change.

## Graphical abstract

Global populations of western mosquitofish *G. affinis* are more sensitive to climatic variation than eastern mosquitofish *G. holbrooki* in terms of length‐weight allometries. Under climate change, the populations of *G. affinis* , especially those in East Asia and Eastern Europe, are expected to grow faster in weight, posing greater threats to native aquatic diversity.

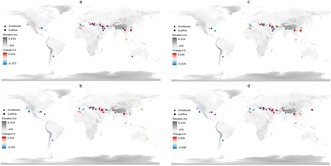

## 1 Introduction

Eastern mosquitofish ( *Gambusia holbrooki* ) and western mosquitofish ( *Gambusia affinis* ) are two *Gambusia* (Poeciliidae) species highly invasive to global aquatic ecosystems (Hurlbert and Mulla 1981; Haynes and Cashner 1995; Pyke 2008; Thompson et al. 2012; Santi et al. 2020). Originated in North America, they were introduced to every continent except Antarctica as early as the 1900s for biological control of mosquito (Gerberich 1946; Krumholz 1948; Azevedo‐Santos et al. 2016; Jourdan et al. 2021). However, as highly productive (Haynes and Cashner 1995; O'dea et al. 2014) and mobile (Rehage and Sih 2004) fish species with strong tolerance to adverse environmental conditions (Alcaraz and García‐Berthou 2007; Pyke 2008) and adaptability to a wide range of habitats (Hinchliffe et al. 2017; Pirroni et al. 2021), they rapidly evolve and expand their distribution (Stearns 1983; Santi et al. 2020). Their invasion threatens endemic fish species, such as Medaka ( *Oryzias latipes* ) in Asia (Tsang and Dudgeon 2021) and Iberian toothcarp (*Apricaphanius iberus*) in Europe (Carmona‐Catot et al. 2013), and other organisms, such as aquatic insects (Merkley et al. 2015) and amphibians (Cabrera‐Guzmán et al. 2017), resulting in a decline in native aquatic diversity and degradation of freshwater ecosystems (Pyke 2008; Xiong et al. 2019). Though efforts have been made to control the invasion of mosquitofish, such as setting barriers (Alemadi and Jenkins 2008) and introducing predators (Cano‐Rocabayera et al. 2019), early eradication seems to be the most feasible and effective way (Cano‐Rocabayera et al. 2019; Kalogianni et al. 2025).

Climate change likely facilitates the spread of mosquitofish in non‐native aquatic habitats (Vondracek et al. 1988; Wilson et al. 2007; Chan et al. 2019; Magellan et al. 2019; Jourdan et al. 2021). In warmer temperatures, it is advantageous for mosquitofish to compete against native fish for their strong adaptive ability (Carmona‐Catot et al. 2013; Magellan et al. 2019). At the population level, the mating success of mosquitofish increases with more thermal acclimation (Wilson et al. 2007), and they breed more in warmer water (Vondracek et al. 1988). In addition to the benefits from warmer water temperatures, higher precipitation seasonality is found to be associated with the expansion of mosquitofish to non‐native habitats, likely due to an increase in the abundance of mosquito larvae and other food sources (Jourdan et al. 2021). At the individual level, warming likely enlarges the body size and improves the body condition of small‐sized fish, including mosquitofish (Daufresne et al. 2009; Lefevre et al. 2017; Audzijonyte et al. 2020). Evidence from transcriptome analyses shows that the individuals living in warmer temperatures demonstrate adaptive evolution (Xie et al. 2019). Mobility, exploration, foraging, and learning abilities of mosquitofish living in warmer temperatures also increase (Magellan et al. 2019). In addition, faster wind speed and stronger fluctuations in water promote the survival and development of mosquitofish (Casamitjana et al. 2019). Among variations in body conditions, behaviors, life history, and population dynamics, change in body size is a common pattern in the mosquitofish populations that demonstrate strong competitiveness against native fish (Haynes and Cashner 1995; Daufresne et al. 2009; Lefevre et al. 2017), which can be estimated based on their length‐weight relationship (Ricker 1973; Jones et al. 1999).

Length‐weight relationship is a common allometric relationship in living organisms from zooplankton (Uye 1982), fish (Ricker 1973; Jones et al. 1999), amphibians (Santini et al. 2018) and reptiles (Meiri 2010) to mammals (Amaral et al. 2010). It commonly follows the power law between weight (*W*; g) and length (*L*; mm) as *W* = *aL* ^*b*^, where the parameter *a* is a constant and *b* is the exponent of the length‐weight allometry (Safran 1992; Jones et al. 1999). The length‐weight relationship can be used to assess the body condition (Jones et al. 1999), fecundity (Dadzie and Wangila 1980), movement (Hansen and Closs 2009), and invasiveness (Rypel 2014) of fish. In fish, if the value of the scaling exponent *b* equals 3, its growth is isometric with a stable proportion between body length and weight (Cone 1989; Jones et al. 1999; Karachle and Stergiou 2012). If the value of the scaling exponent *b* is above 3, the fish growth is allometric and it grows faster in weight than in length, indicating optimum environmental conditions for its mobility and competitiveness (Mitz and Newman 1989; Jones et al. 1999; Karachle and Stergiou 2012). In the origins of mosquitofish, that is, North America, it has been the subject of studies on fish biology, community ecology, and ecological applications (Krumholz 1948; Brown and Fox 1966; Brown‐Peterson and Peterson 1990), including its length‐weight relationship (Klassen et al. 2014). In other countries where it rapidly invades non‐native habitats, a number of studies reported its occurrence and length‐weight relationship (Eagderi and Radkhah 2015; Kurtul and Sari 2020; Xiong et al. 2020; Hashimoto et al. 2024). Although studies show that warming can enlarge the body size and improve the body condition of mosquitofish at the lab (Vondracek et al. 1988; Carmona‐Catot et al. 2013), it remains unclear how the length‐weight allometries of its global populations might vary under climate change. This study compiled a global dataset of the length‐weight allometry of mosquitofish and tested the effects of climate on the values of the scaling exponent *b* of global populations of mosquitofish. The results will highlight the need for early identification and control of invasive mosquitofish in the non‐native habitats under climate change.

## 2 Methods

### 2.1 Allometry Data

**FIGURE 1:**
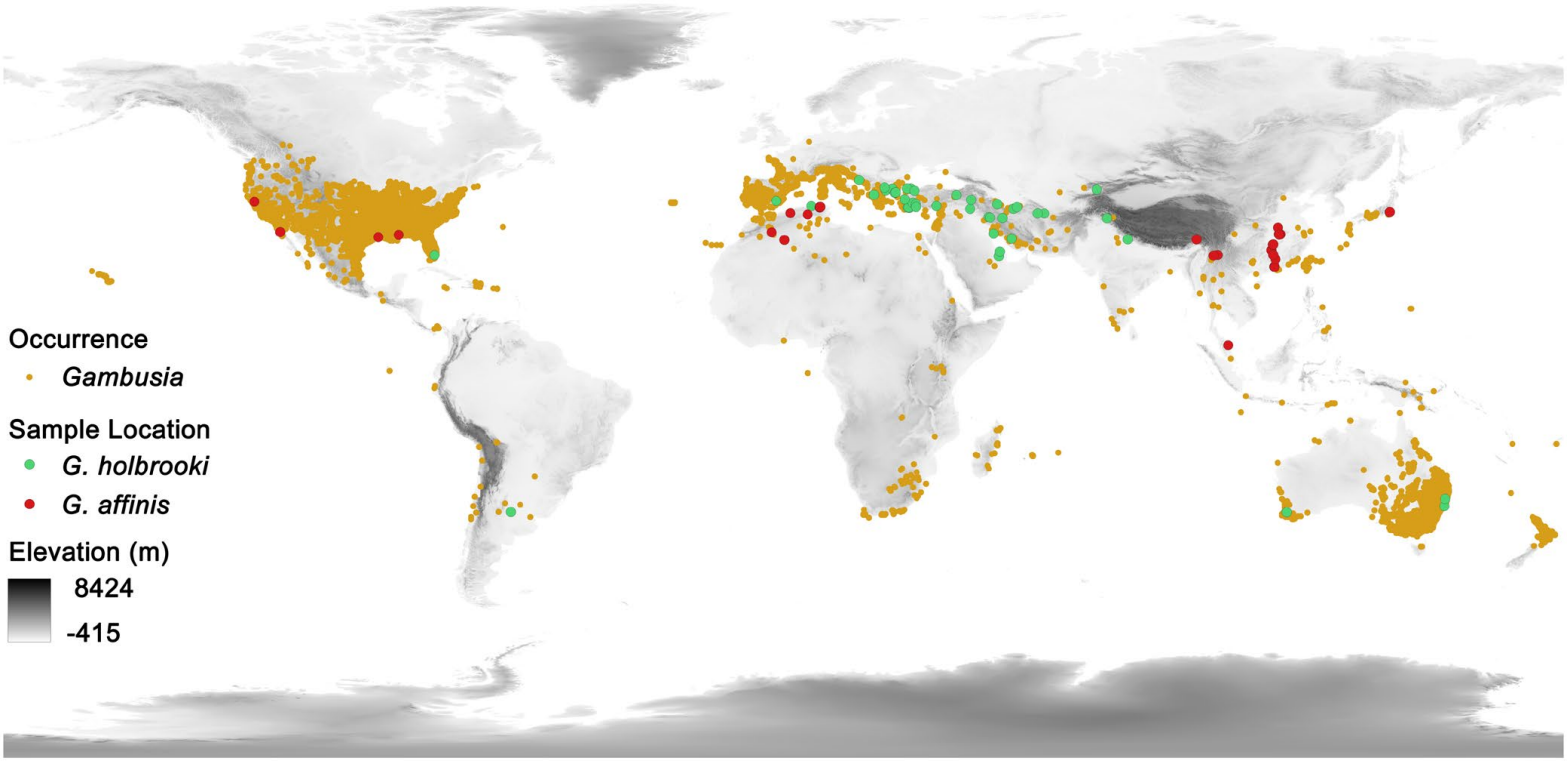
Distribution of the invasive mosquitofish (in yellow) and locations of the 79 samples (45 of *G. holbrooki* in green and 34 of *G. affinis* in red). The base map shows the elevation of global surface derived from the STRM DEM product.

We focused on the populations of mosquitofish distributed in natural environments across the globe (Figure 1). We searched literature published from 1960 using the keywords (“mosquitofish” OR “ *Gambusia holbrooki* ” OR “ *Gambusia affinis* ” OR “ *G. holbrooki* ” OR “ *G. affinis* ”) AND (“length‐weight relationship” OR “allometry”) for the studies on the length‐weight relationships of mosquitofish from Web of Science (WOS), JSTOR, Google Scholar, and China National Knowledge Infrastructure (CNKI). The languages of literature include English, French, Spanish, Arabic, Hindi, Chinese, Japanese, and Korean, which correspond to the official languages for scientific publications in the countries where mosquitofish distributes. From 2050 articles, we found 49 nonduplicate studies that report the length‐weight relationships of 66 populations of *G. holbrooki* or *G. affinis* with the minimum sample size of 50 individuals (Figure 1). A large sample size reduces estimation error in the parameters of the allometric model (De Robertis and Williams 2008).

In addition to the allometry data for the populations from 66 locations (Figure 1), we estimated the length‐weight relationship (*W* = *aL* ^*b*^) based on the weight (g) and total length (mm) of all adults of each population (Ricker 1973) collected from 12 locations in southern and central China between 2015 and 2016 and a location in western China in 2020 (Figure 1) using the nls function in R 4.4.3 (Ritz and Streibig 2008). We then compiled the estimated values of the parameter *b* of the length‐weight relationship of each population, together with the coordinates of its location. In the cases where the exact coordinates are not available in the articles, we used Google Earth to locate the sample sites based on the descriptions of methods. Elevation of each site was extracted from 30‐s SRTM Digital Elevation Model (DEM) based on its coordinates (Yang et al. 2011). In total, we compiled the values of the scaling exponent *b* of 79 mosquitofish populations in 17 countries of all continents except Antarctica (Table 1). The year of samples ranged from 1990 to 2024, with the mean of 2012. We examined the distribution of the values of the scaling exponent b and tested if the mean values of the parameter *b* are equal to or larger than 3 using *t* tests.

**TABLE 1:** Summary of the coordinates and values of the scaling exponent *b* of the 79 populations of mosquitofish by continents.

Continent	Species (*n*)	Latitude (°)	Longitude (°)	Elevation (m)	*b*
North America	*G. holbrooki* (1)	25.75	−80.56	6	3.020
	*G. affinis* (4)	29.90–38.08 (32.37)	−122.11 to −88.80 (−105.15)	2–81 (23)	3.081–3.450 (3.263)
South America	*G. holbrooki* (1)	−33.42	−62.90	113	3.310
	*G. affinis* (0)	—	—	—	—
Europe	*G. holbrooki* (7)	37.05–43.05 (40.14)	−1.68 to 25.13 (16.45)	0–543 (207)	2.952–3.590 (3.286)
	*G. affinis* (0)	—	—	—	—
Africa	*G. holbrooki* (0)	—	—	—	—
	*G. affinis* (7)	29.25–36.87 (34.47)	−2.72 to 8.50 (4.28)	−2–928 (323)	2.950–3.200 (3.110)
Asia	*G. holbrooki* (33)	25.49–41.07 (36.15)	25.71–79.46 (42.88)	−31–2044 (638)	2.680–3.763 (3.229)
	*G. affinis* (23)	5.00–35.72 (26.83)	95.35–139.99 (113.42)	1–1962 (232)	2.846–3.460 (3.175)
Oceania	*G. holbrooki* (3)	−33.47 to −30.43 (−31.99)	116.21–152.73 (140.48)	10–298 (130)	3.142–3.168 (3.152)
	*G. affinis* (0)	—	—	—	—
Global	*G. holbrooki* (45)	−33.47–43.05 (30.45)	−80.56–152.73 (40.18)	−31–2044 (511)	2.680–3.763 (3.230)
	*G. affinis* (34)	5.00–38.08 (29.06)	−122.11–139.99 (65.23)	−2–1962 (226)	2.846–3.460 (3.172)

*Note:* The number of sample (*n*) as 0 does not necessarily mean that the species does not distribute in that continent. Mean latitude, longitude, elevation and value of the scaling exponent *b* are in the bracket after the range.

### 2.2 Climate Data

The 79 populations distributed in a variety of climate zones, including arid desert and steppe, tropical rainforest and savannah, and five temperate zones (Cui et al. 2021). We initially extracted 24 historical climatic variables at each location from the 30‐s WorldClim 2.1 data for 1970–2000 (Fick and Hijmans 2017) together with aridity index (AI) and reference evapotranspiration (ET_0_) from the 30‐s Global AI PET database for the same period (Zomer et al. 2022). We examined the correlation (r) between each pair of these 26 variables using the R package SpatialPack (Vallejos et al. 2020) based on the modified *t* test for spatial processes (Dutilleul et al. 1993), and kept 11 of those with *r* < 0.8 upon screening the potential multicollinearity (Table S1). We then used the R PerformanceAnalytics package to visualize the correlation between each pair of the explanatory variables (Figure S1).

### 2.3 Regression Models

We applied both statistical and machine learning methods to build spatial regression models for estimating the values of the scaling exponent *b* of *G. holbrooki* and those of *G. affinis* . The statistical models are useful for inferring the potential relationships between climate and the length‐weight allometry, while machine learning methods are helpful to identify complex nonlinear relationships (Bzdok et al. 2018). Both methods address the potential spatial autocorrelation within the global populations (Breiman 2001; Pinheiro et al. 2007).

First, we built generalized least squares models using the R nlme package (Pinheiro et al. 2007) with year of sample, elevation, and the 11 bioclimatic variables as the explanatory variables. While building the generalized least squares models, we added the second order terms of these explanatory variables in case of nonlinear relationships and accounted for the spatial autocorrelation in the values of the scaling exponent *b* with the correlation structure corSpatial (Pinheiro et al. 2007). We then used the stepAIC function based on the Akaike information criterion (AIC) to conduct stepwise selection for the best model (Zhang 2016). Continent was considered as the random effect of the model, but no decrease in AIC was achieved by including this random term. The variance inflation factor (VIF) of each explanatory variable of the parameterized model was calculated to examine if it was below 5, indicating that collinearity was not a concern of the model (O'brien 2007).

Second, we built random forest regression models (Breiman 2001) with the same 13 explanatory variables as predictors to assess the effects of climate on the values of the scaling exponent *b* using the R randomForest package (Liaw and Wiener 2002). No second order term of the explanatory variable was added as the random forest algorithm accounts for potential nonlinear patterns (Breiman 2001). The explanatory variables were selected based on the averaged variable importance and predictive accuracy through the 10‐fold cross validation using the R steprf package (Li 2022), which avoided overfitting with less important explanatory variables in the parameterized random forest regression model (Speiser et al. 2019). Partial dependence of the scaling exponent *b* on each important explanatory variable is visualized using the R pdp package (Greenwell 2017). Compared with the generalized least squares model, the random forest regression model is useful for modeling complex relationships among spatial variables (Hengl et al. 2018).

### 2.4 Projection

Based on the parameterized generalized least squares models and the random forest regression models, we projected the values of the scaling exponent *b* of the 79 mosquitofish populations under two climate scenarios in the 2050s by fitting projected climate data while assuming other conditions would remain unchanged. The two scenarios are Shared Socioeconomic Pathways (SSPs) 245 and 585, which project future climate based on different scenarios of CO_2_ emissions (O'Neill et al. 2014). SSP 245 sets a moderate reduction in emissions while SSP 585 sets more realistic emission targets with a larger quota on emissions (O'Neill et al. 2014). The future climate projections for the period 2041–2060 at the locations of the 79 populations were extracted as the means of the future climatic variables projected by 11 30‐s CMIP6 global climate models (GCMs) available from WorldClim v2.1 (Fick and Hijmans 2017).

## 3 Results

### 3.1 Distribution of the Scaling Exponent

**FIGURE 2:**
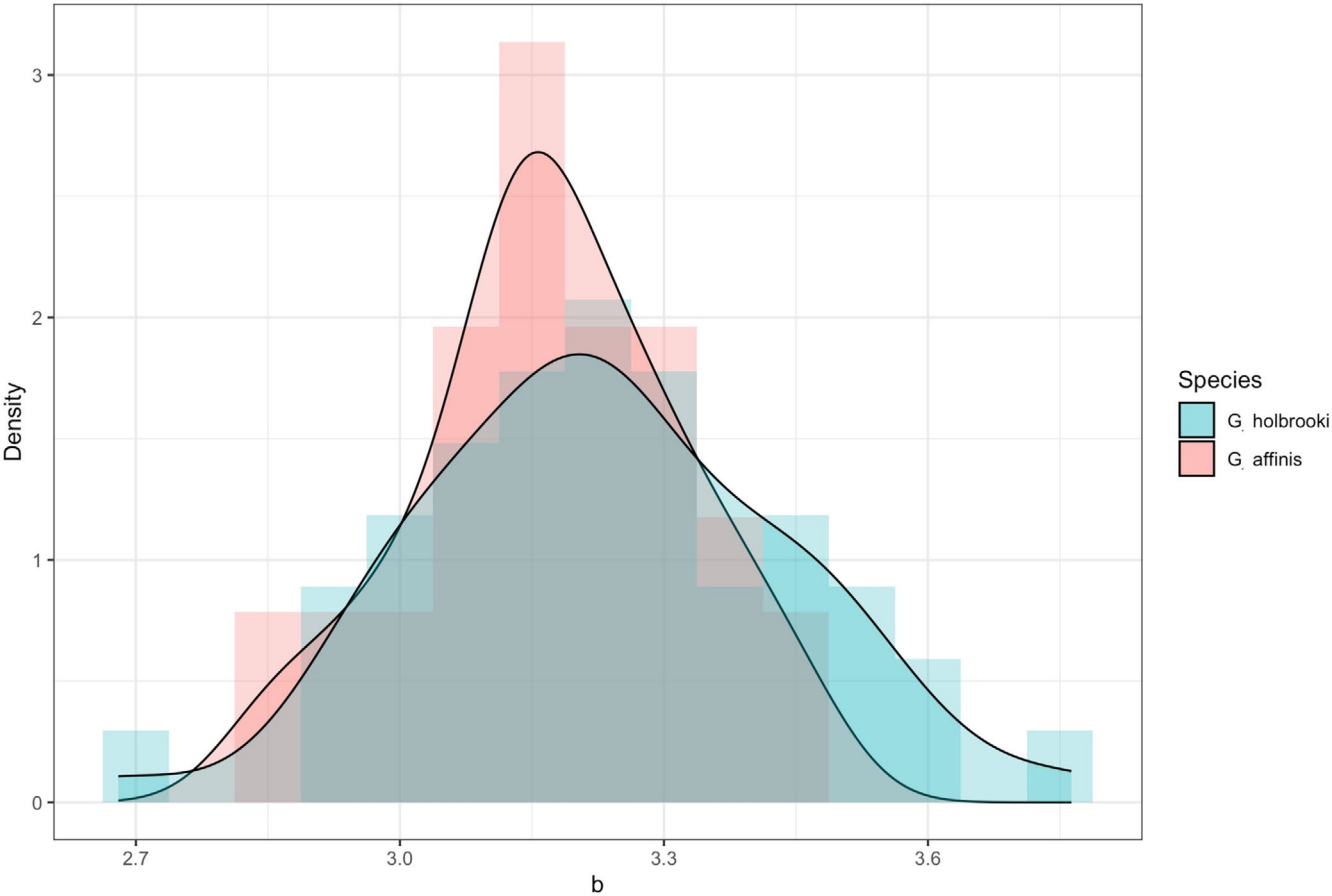
Probability density distribution of the values of the scaling exponent *b* of the length‐weight relationship among *G. holbrooki* (blue) and *G. affinis* (red). The black curves show the kernel density estimates of the values of the scaling exponent *b* of the two species.

The values of the scaling exponent *b* of the length‐weight relationship among the 79 populations range from 2.68 to 3.76 with the mean of 3.21 and the standard deviation of 0.19 (Table S1). Mean (±standard deviation) of the values of the scaling exponent *b* of the 45 populations of *G. holbrooki* and that of the 34 populations of *G. affinis* are 3.23 (±0.21) and 3.17 (±0.15), respectively (Figure 2). The mean values of the scaling exponent *b* of the two mosquitofish species are significantly higher than 3 (*p* < 10^−14^ for each *t* test).

### 3.2 Generalized Least Squares Model

The best selected generalized least squares models for *G. holbrooki* and *G. affinis* have different sets of the explanatory variables (Table 2). The model for *G. holbrooki* (1) includes the second order of mean annual temperature but that for *G. affinis* (2) does not include any second order term. The VIF of each variable is below 5:

(1) $$\begin{array}{cc} {\hat{b}}_{G.\text{holbrooki}}= & 7.38\times 10^{-1}+8.89\times 10^{-2}\text{Tavg}-5.62\times 10^{-3}{\text{Tavg}}^{2}\\ & -1.71\times 10^{-1}\mathrm{TD}+3.06\times 10^{-2}\mathrm{BIO}03+8.62\\ & \times 10^{-2}\mathrm{BIO}05-6.07\times 10^{-4}\mathrm{BIO}19+4.18\times 10^{-4}\mathrm{ET}0, \end{array}$$

(2) $$\begin{array}{cc} {\hat{b}}_{G.\text{affinis}}= & 4.91-3.25\times 10^{-4}\text{Elev}-2.31\times 10^{-4}\text{Prec}\\ & -1.65\times 10^{-1}\text{Wind}-7.64\times 10^{-3}\mathrm{BIO}03\\ & -7.33\times 10^{-4}\mathrm{BIO}04-3.33\times 10^{-3}\mathrm{BIO}15, \end{array}$$

where ${\hat{b}}_{G.\text{holbrooki}}$ and ${\hat{b}}_{G.\text{affinis}}$ are the predicted scaling exponents of *G. holbrooki* and *G. affinis* , respectively. The explanatory variables include elevation (Elev), mean annual temperature (Tavg), difference between annual maximum and minimum temperature (TD), annual precipitation (Prec), wind speed (Wind), isothermality (BIO03), temperature seasonality (BIO04), maximum temperature of warmest month (BIO05), mean temperature of wettest quarter (BIO08), precipitation seasonality (BIO15), precipitation of coldest quarter (BIO19), and annual reference evapotranspiration (ET0).

**TABLE 2:** Estimated coefficients of the best selected generalized least squares models for *G. holbrooki* and *G. affinis* .

	Estimate	SE	*t* value	*p*
*G. holbrooki*				
Intercept	7.38 × 10^−1^	9.04 × 10^−1^	0.816	0.420
Mean annual temperature	8.89 × 10^−2^	6.74 × 10^−2^	1.319	0.195
Mean annual temperature^2^	−5.62 × 10^−3^	1.84 × 10^−3^	−3.053	0.004
Difference between annual maximum and minimum temperature	−1.71 × 10^−1^	7.37 × 10^−2^	−2.325	0.026
Isothermality	3.06 × 10^−2^	1.89 × 10^−2^	1.622	0.113
Maximum temperature of warmest month	8.62 × 10^−2^	4.58 × 10^−2^	1.881	0.068
Precipitation of coldest quarter	−6.07 × 10^−4^	2.73 × 10^−4^	−2.224	0.032
Annual reference evapotranspiration	4.18 × 10^−4^	2.04 × 10^−4^	2.052	0.047
*G. affinis*				
Intercept	4.91	5.29 × 10^−1^	9.272	< 0.001
Elevation	−3.25 × 10^−4^	6.50 × 10^−5^	−5.001	< 0.001
Annual precipitation	−2.31 × 10^−4^	7.45 × 10^−5^	−3.097	0.005
Wind speed	−1.65 × 10^−1^	4.12 × 10^−2^	−3.996	< 0.001
Isothermality	−7.64 × 10^−3^	4.32 × 10^−3^	−1.771	0.088
Temperature seasonality	−7.33 × 10^−4^	3.03 × 10^−4^	−2.419	0.023
Precipitation seasonality	−3.33 × 10^−3^	1.85 × 10^−3^	−1.803	0.083

*Note:* Standard error (SE) of the estimated coefficient, and *t* value and *p* value of the *t* test on the coefficient are listed in Table 2. The AIC for the model for *G. holbrooki* is 69.2 and that for *G. affinis* is 45.0.

The estimated effect of mean annual temperature on the scaling exponent *b* becomes negative after mean annual temperature exceeds 15.82°C. The difference between annual maximum and minimum temperature is negatively associated with the scaling exponent *b* of *G. holbrooki* . Precipitation of the coldest quarter is also negatively associated with the scaling exponent *b* of *G. holbrooki* , while annual reference evapotranspiration is positively associated with it. For *G. affinis* , elevation, annual precipitation, wind speed, and temperature seasonality are negatively associated with the scaling exponent *b*.

### 3.3 Random Forest Model

**FIGURE 3:**
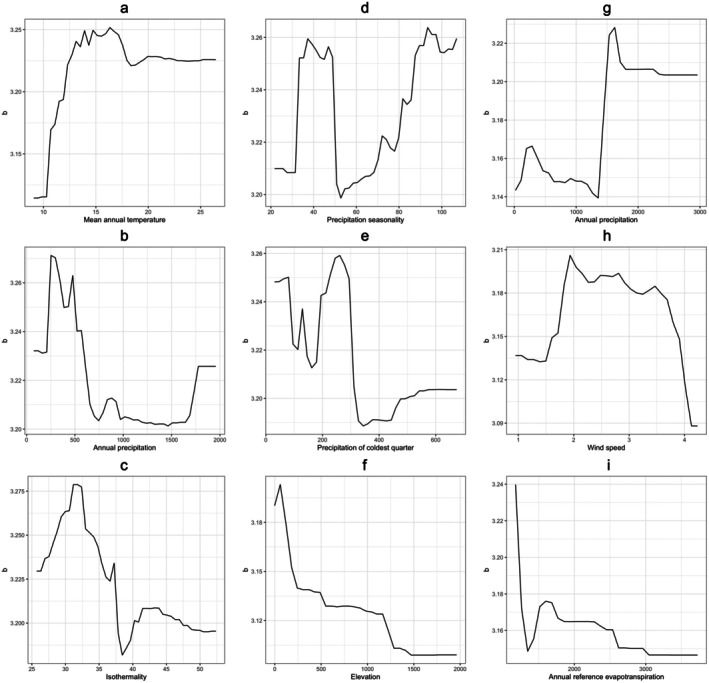
Partial dependence of the scaling exponent *b* on each of mean annual temperature (a), annual precipitation (b), isothermality (c), precipitation seasonality (d), and precipitation of coldest quarter (e) of the random forest regression model for *G. holbrooki* and that on each of elevation (f), annual precipitation (g), wind speed (h), and annual reference evapotranspiration (i) of the model for *G. affinis* .

The random forest regression models with the lowest prediction error for the scaling exponent *b* are selected for the two mosquitofish species. The variables included for the most accurate random forest regression model for *G. holbrooki* are mean annual temperature, annual precipitation, isothermality, precipitation seasonality, and precipitation of coldest quarter (Figure S2a,b). Meanwhile, those for *G. affinis* are elevation, annual precipitation, wind speed, and annual reference evapotranspiration (Figure S2c,d). Based on the most accurate random forest regression models, partial dependence of the scaling exponent *b* on the explanatory variables for *G. holbrooki* (Figure 3a–e) and *G. affinis* (Figure 3f–i) populations show similar relationships as identified from the generalized least square models. These partial dependence plots illustrate the effect of individual explanatory variables while averaging the effects of the other variables (Figure 3), which should be interpreted with caution for the potential interaction effects.

### 3.4 Projection for the Scaling Exponent

**FIGURE 4:**
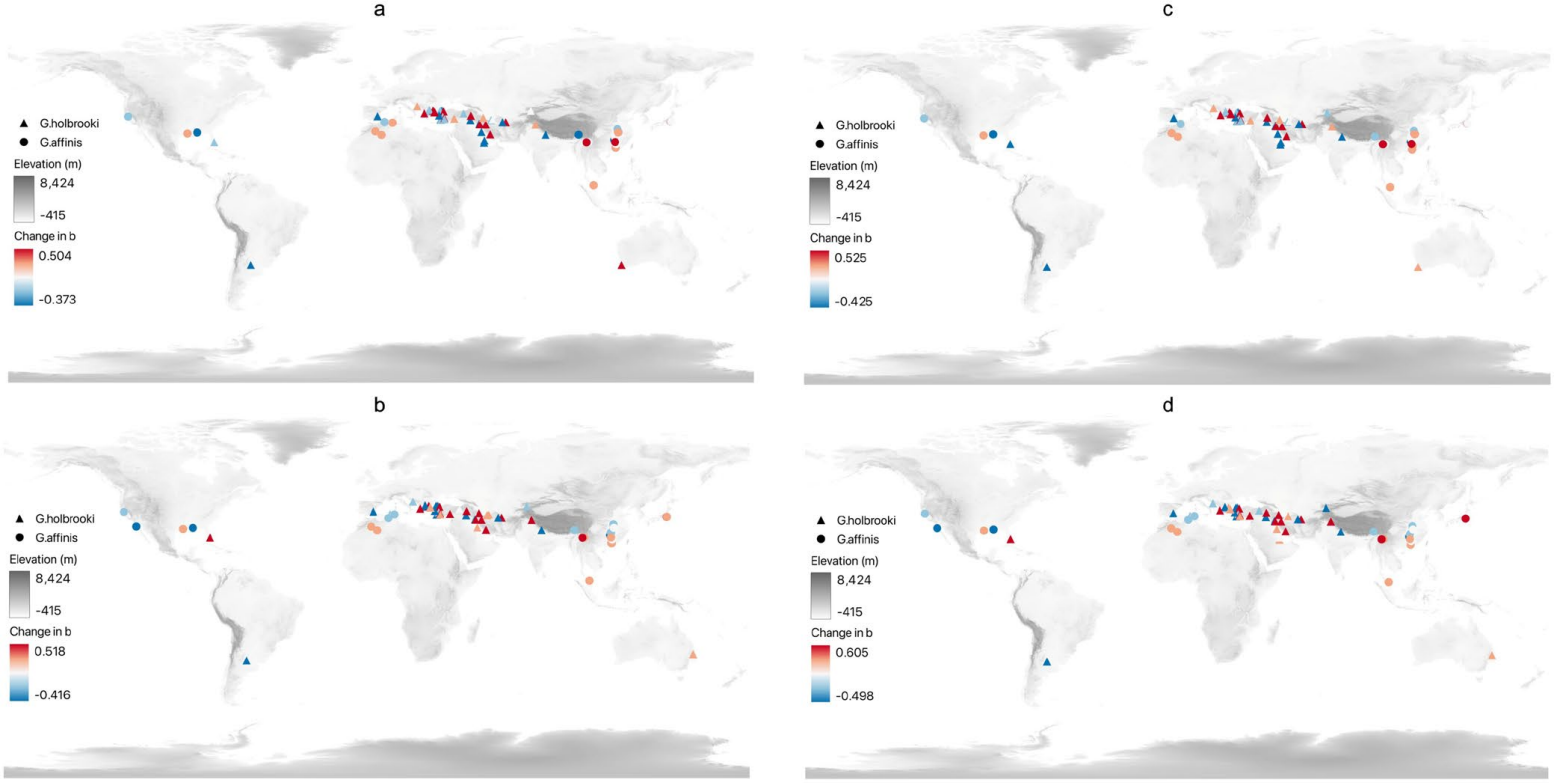
Changes in the scaling exponent *b* of the 79 mosquitofish populations (45 triangles for *G. holbrooki* and 34 circles for *G. affinis* ) across the globe in the 2050s under SSP 245 (a and b) and SSP 585 (c and d) climate change scenarios. The projected changes are based on the selected generalized least square model (a and c) and the best random forest regression model (b and d). Positive changes are in the yellow‐to‐red gradient and negative changes are in the light‐to‐dark blue gradient. The base map is the terrain elevation (m).

Changes in the values of the scaling exponent *b* among the 79 populations of the two mosquitofish species across the globe are projected under SSP 245 (Figure 4a,b) and SSP 585 (Figure 4c,d) climate change scenarios in the 2050s based on the selected generalized least square models (Figure 4a,c) and the most accurate random forest regression models (Figure 4b,d). There is an agreement in the distribution of changes in the scaling exponent *b* between the present and the projections under the two climate change scenarios, though the magnitudes of projected changes vary (Figure 4a vs. c by the generalized least square model and Figure 4b vs. d by the random forest regression model). Disagreement between the projections by the two models occurs in the directions of changes in the scaling exponent *b* among the populations in the Middle East (Figure 4a vs. b under SSP 245 and Figure 4c vs. d under SSP 585). An increase in the scaling exponent *b* is projected to occur among the populations in East Asia, Oceania, Eastern Europe, and North Africa, while a decrease in the scaling exponent *b* is projected to occur among the populations in South Asia, the Middle East, Western Europe, North America, and South America.

## 4 Discussion

This study quantifies the effect of climate on the length‐weight relationships of the invasive mosquitofish populations across the globe (Figure 1). The mean value of the scaling exponent *b* of the 45 populations of *G. holbrooki* and that of 34 populations of *G. affinis* is significantly higher than 3, though the variation in the scaling exponent *b* across the globe is large (Figure 2). The statistical and machine learning models used in this study identify similar relationships between the climatic variables and the scaling exponent *b* (Table 2 and Figure 3). Nonlinear relationships between mean annual temperature and the scaling exponent *b* are identified from the selected generalized least square model for *G. holbrooki* , which is also captured by the random forest regression model (Figure 3a). The fitted generalized least square model indicates that *G. holbrooki* populations habituating in warmer regions, given that mean annual temperature is above 15.82°C, will grow less weight than those in colder regions of the same lengths, which could be due to elevated metabolism and reduced food availability in the warmer aquatic environment (Paloheimo and Dickie 1966; De Almeida‐Val et al. 2005). However, the complex relationships between precipitation seasonality (Figure 3d) and the scaling exponent *b* for *G. holbrooki* and between annual precipitation and the scaling exponent *b* for *G. affinis* (Figure 3g) are not included in the selected generalized least square models (Table 2). The statistical models are easier to interpret while the machine learning methods can reveal more complex relationships between climate and the scaling exponent *b* of mosquitofish. It would be ideal to combine these modeling approaches when estimating the potential effects of climate on the length‐weight relationships (Xu et al. 2021; Flores et al. 2024).

Besides, elevation, evapotranspiration, wind speed, precipitation, and seasonality are the other variables found associated with the scaling exponent *b*. For the populations of *G. affinis* habituating at the higher altitudes, they grow less weight than those at the lower altitudes of the same lengths given the other conditions unchanged (Table 2). The decreased oxygen levels, reduced volumes of algae, and shifted composition of aquatic organisms at higher altitudes might change the metabolic and physiological characters of mosquitofish (De Almeida‐Val et al. 2005; Xie et al. 2019; Zhang et al. 2024). For the populations of *G. holbrooki* habituating in the aquatic environments with higher annual reference evapotranspiration, their biomass accumulation could be impacted due to increased groundwater recharge and intensified streamflow (Henriksen et al. 2021) as well as increased salinity (Dendrinos and Thorpe 1985; Brown‐Peterson and Peterson 1990), especially in Turkey, the Middle East, and North Africa (Kurtul and Sari 2020; Zomer et al. 2022). For the populations of *G. affinis* encountering stronger wind, they allocate more energy to moving and foraging than those living under milder wind (Donelan et al. 1992; Casamitjana et al. 2019). In terms of precipitation, limited precipitation, especially in the coldest quarter, may reduce base flows and habitat quality of the water bodies where mosquitofish habituate, which is found negatively associated with fish weight growth (Kelson and Carlson 2019; Jourdan et al. 2021). In addition, the seasonality of temperature is negatively associated with the scaling exponent *b* (Table 2), which might be due to the fact that climate extremes, such as drought, disrupt growth, and phenology of fish (Henriksen et al. 2021; Huang et al. 2021).

Projections of the values of the scaling exponent *b* among the global populations of the two mosquitofish species show spatial variation in the impact of climate change on the length‐weight relationship of this invasive species (Figure 4). Although the magnitudes of change in the scaling exponent *b* projected by the statistical and machine learning models are different, the spatial patterns are similar. The *G. affinis* populations in East Asia and Eastern Europe are projected to grow faster in weight than in length by both models under the SSP 245 and SSP 585 climate change scenarios in the 2050s, indicating stronger body condition and higher risks of invasion of mosquitofish in non‐native habitats in these two regions (Jones et al. 1999; Rypel 2014; Eagderi and Radkhah 2015; Pazianoto et al. 2016). Although the existing niche‐based species distribution model for *G. affinis* in China shows that less than 5% of the land of China is suitable for mosquitofish (Cheng et al. 2018; Han et al. 2024), our models indicate that higher risks of invasion by mosquitofish are expected in China under both moderate and severe climate change scenarios (Figure 4). Notably, *G. affinis* populations were recently recorded in the Eastern Himalayas and Tibetan Plateau for the first time (Wang et al. 2023), which may threaten the fish species endemic to these habitats, such as *O. latipes* (Tsang and Dudgeon 2021). In Eastern Europe, faster weight growth than length growth of *G. affinis* is likely to increase the risk of extinction of endemic species and rare aquatic species (Carmona‐Catot et al. 2013; Pirroni et al. 2021).

In addition to climate change, changes in the aquatic environment, including salinity (Dendrinos and Thorpe 1985; Brown‐Peterson and Peterson 1990), hydrological conditions (Chan et al. 2019; Xiong et al. 2019), levels of chemical pollutants (Rawson et al. 2009; Saaristo et al. 2019), aquatic pathogens (Okon et al. 2024), and types of land use near the water bodies (Lee et al. 2017), as well as genetic variation (Abdelnour et al. 2024) and modulation (Wang et al. 2025), may also alter the allometry of mosquitofish. The models built in this study could be improved by incorporating habitat conditions and human activities if data were available (Tan et al. 2025). However, not every study on the length–weight relationship of mosquitofish recorded habitat conditions or collected water samples for laboratory tests (Xiong et al. 2015; Xiong et al. 2020). Also, few studies considered the effects of human activities, such as deforestation, afforestation, and urbanization (Liu et al. 2025), on the length‐weight relationship of mosquitofish. Future studies on the impact of global change on the length‐weight allometries of fish could consider these factors in addition to climate. Moreover, additional populations of mosquitofish across the globe (Figure 1) should be measured, whose length‐weight relationships as well as other biological characteristics, such as metabolism and productivity, should be studied. Genetic studies on global populations of the two mosquitofish species are helpful to understand the mechanisms of their invasive ability and fast adaptation to climate change (Xie et al. 2019; Abdelnour et al. 2024; Zheng et al. 2025). Considering uncertainty in the impacts of climate change on the length‐weight relationships of the two mosquitofish species and their distributions across the globe, it is necessary to frequently monitor their potential habitats, report new occurrences of the invasive populations, and control them from further spread to non‐native habitats.

## Author Contributions

**Shanshan Rao:** data curation (equal), formal analysis (equal), investigation (equal), methodology (equal), validation (equal), visualization (equal), writing – original draft (equal). **Wenjing Qi:** data curation (equal), formal analysis (equal), investigation (equal), methodology (equal), validation (equal), writing – original draft (equal). **Haochen Cao:** data curation (equal), formal analysis (equal), investigation (equal), validation (equal), visualization (equal), writing – original draft (equal). **Cong Tang:** data curation (equal), formal analysis (equal), investigation (equal), methodology (equal), writing – original draft (equal). **Yujie Xiao:** data curation (equal), formal analysis (equal), investigation (equal), methodology (equal), visualization (equal), writing – original draft (equal). **Yuxin Sun:** data curation (equal), formal analysis (equal), investigation (equal), validation (equal), visualization (equal), writing – original draft (equal). **Wen Xiong:** data curation (equal), investigation (equal), methodology (equal), project administration (equal), supervision (equal), validation (equal), writing – original draft (equal). **Peng Xie:** funding acquisition (equal), investigation (equal), project administration (equal), resources (equal), supervision (equal), validation (equal), writing – original draft (equal), writing – review and editing (equal). **Kun Xu:** conceptualization (equal), funding acquisition (equal), methodology (equal), supervision (equal), validation (equal), writing – original draft (equal), writing – review and editing (equal).

## Funding

This study is supported by the Third Xinjiang Scientific Expedition (grand number 2022xjkk0204), the National Natural Science Foundation of China (grant numbers 52179083 and 32002390), and the Department of Education of Hubei Province (grant number B2024129).

## Conflicts of Interest

The authors declare no conflicts of interest.

## Supporting information


**Table S1:** Summary of the 11 climatic variables of the locations where the 79 mosquitofish samples were collected. Range, mean and standard deviation (SD) of each variable are listed for the two mosquitofish species.
**Figure S1:** Correlation between each pair of the scaling exponent *b*, elevation (Elev) and the 11 bioclimatic variables. Pearson correlation coefficients (*r*) at the top right panels and scatterplots with LOESS smoothing lines at the bottom left panels with distributions of the variables in diagonal. The 11 bioclimatic variables are mean annual temperature (Tavg), difference between annual maximum and minimum temperature (TD), annual precipitation (Prec), wind speed (Wind), isothermality (BIO03), temperature seasonality (BIO04), maximum temperature of warmest month (BIO05), mean temperature of wettest quarter (BIO08), precipitation seasonality (BIO15), precipitation of coldest quarter (BIO19), and annual reference evapotranspiration (ET0).
**Figure S2:** Variable importance plots for the best random forest regression models for the two mosquitofish species. Mean annual temperature (Tavg), annual precipitation (Prec), isothermality (BIO03), precipitation seasonality (BIO15), and precipitation of coldest quarter (BIO19) are the predictors for the model for *G. holbrooki* (a and b). Elevation (Elev), annual precipitation (Prec), wind speed (Wind), and annual reference evapotranspiration (ET0) are the predictors for *G. affinis* (c and d). %IncMSE is the percentage of increase in mean square error if the value of this variable is randomly assigned (a and c). IncNodePurity is increase in node purity if the variable is added to the model (b and d).

## Data Availability

Data are available via figshare at https://doi.org/10.6084/m9.figshare.30671531.v1.
