# Supplementary material for: Faster Weight Growth in Invasive Mosquitofish Gambusia holbrooki and Gambusia affinis (Poeciliidae) Under Climate Change
Source: Ecol Evol. 2026 Jan 12;16(1):e72943. doi: 10.1002/ece3.72943 (PMC12796510; doi:10.1002/ece3.72943)
Supplement: Supplementary file 1 — Table S1: Summary of the 11 climatic variables of the locations where the 79 mosquitofish samples were collected. Range, mean and standard deviation (SD) of each variable are listed for the two mosquitofish species. Figure S1: Correlation between each pair of the scaling exponent b, elevation (Elev) and the 11 bioclimatic variables. Pearson correlation coefficients (r) at the top right panels and scatterplots with LOESS smoothing lines at the bottom left panels with distributions of the variables in diagonal. The 11 bioclimatic variables are mean annual temperature (Tavg), difference between annual maximum and minimum temperature (TD), annual precipitation (Prec), wind speed (Wind), isothermality (BIO03), temperature seasonality (BIO04), maximum temperature of warmest month (BIO05), mean temperature of wettest quarter (BIO08), precipitation seasonality (BIO15), precipitation of coldest quarter (BIO19), and annual reference evapotranspiration (ET0). Figure S2: Variable importance plots for the best random forest regression models for the two mosquitofish species. Mean annual temperature (Tavg), annual precipitation (Prec), isothermality (BIO03), precipitation seasonality (BIO15), and precipitation of coldest quarter (BIO19) are the predictors for the model for G. holbrooki (a and b). Elevation (Elev), annual precipitation (Prec), wind speed (Wind), and annual reference evapotranspiration (ET0) are the predictors for G. affinis (c and d). %IncMSE is the percentage of increase in mean square error if the value of this variable is randomly assigned (a and c). IncNodePurity is increase in node purity if the variable is added to the model (b and d). [file ECE3-16-e72943-s001.docx]

**Supplementary Materials**

**Table S1** Summary of the 11 climatic variables of the locations where the 79 mosquitofish samples were collected. Range, mean and standard deviation (SD) of each variable are listed for the two mosquitofish species.

|  | *G. holbrooki* | | *G. affinis* | |
| --- | --- | --- | --- | --- |
| Variable (Unit) | Range | Mean (SD) | Range | Mean (SD) |
| Mean annual temperature (°C) | 9.1 – 26.5 | 16.0 (3.9) | 15.2 – 25.7 | 18.8 (2.9) |
| Difference between annual maximum and minimum temperature (°C) | 6.6 – 15.6 | 10.7 (2.3) | 7.1 – 15.4 | 9.4 (2.4) |
| Annual precipitation (mm) | 74 – 1957 | 662 (395) | 20 – 2960 | 1208 (588) |
| Wind speed (m/s) | 0.93 – 4.37 | 2.61 (0.75) | 0.95 – 4.23 | 2.54 (0.93) |
| BIO3 Isothermality (100 × mean diurnal range/temperature annual range) | 25.7 – 52.4 | 36.1 (6.4) | 23.0 – 74.9 | 34.9 (11.3) |
| BIO4 Temperature seasonality (standard deviation × 100) | 339.4 – 1029.4 | 722.3 (163.7) | 82.2 – 921.5 | 686.4 (196.5) |
| BIO5 Maximum temperature of warmest month (°C) | 24.0 – 43.6 | 31.9 (4.5) | 24.4 – 46.6 | 32.7 (3.8) |
| BIO8 Mean temperature of wettest quarter (°C) | 2.9 – 27.7 | 10.3 (5.6) | 7.9 – 27.6 | 21.7 (5.9) |
| BIO15 Precipitation seasonality (coefficient of variation of monthly precipitation) | 21.7 – 107.0 | 64.9 (23.6) | 17.4 – 94.3 | 57.0 (16.4) |
| BIO19 Precipitation of coldest quarter (mm) | 31 – 674 | 229 (142) | 6 – 910 | 196 (165) |
| Annual reference evapotranspiration (mm) | 934 – 3025 | 1691 (421) | 1174 – 3728 | 1514 (520) |


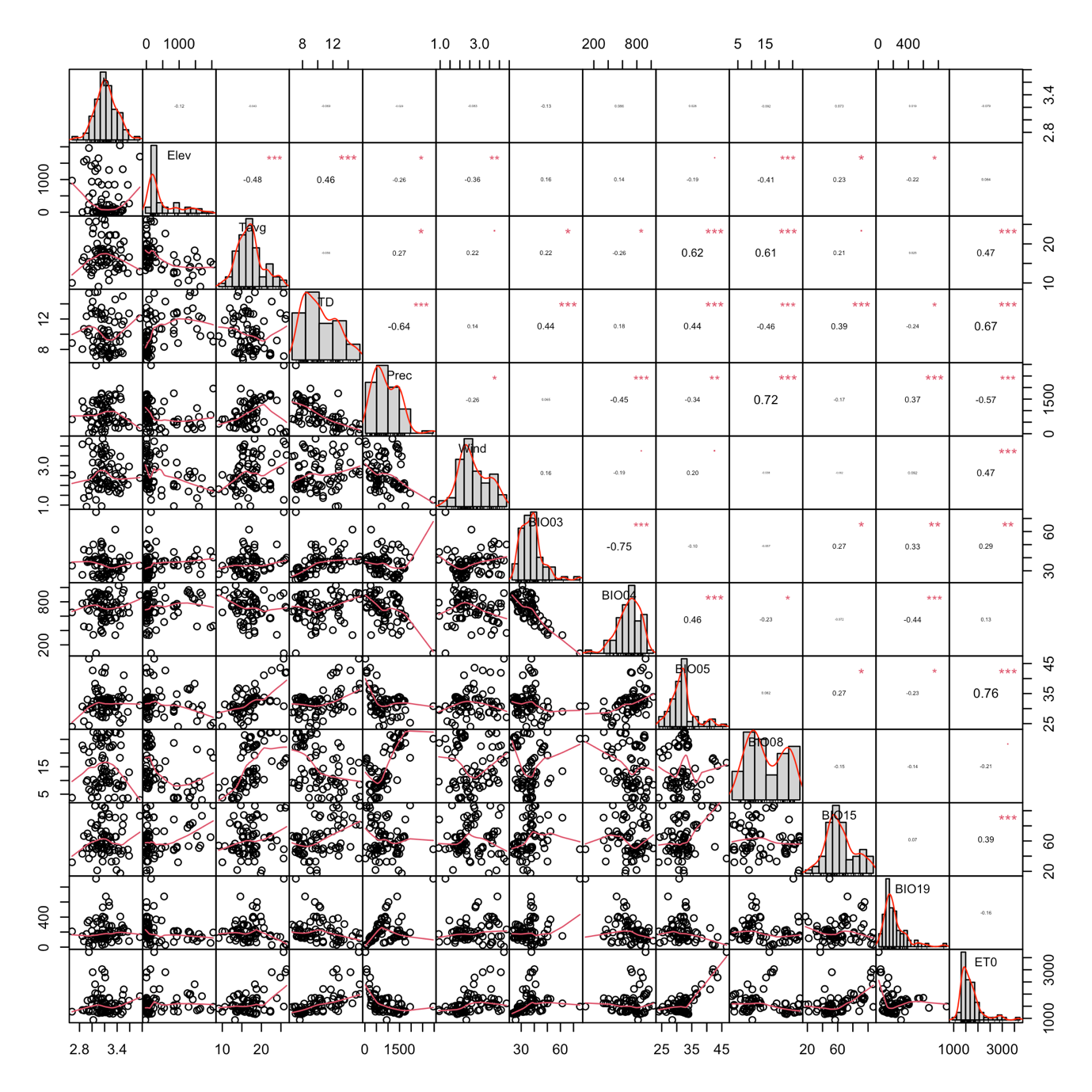


**Figure S1** Correlation between each pair of b, elevation (Elev) and the 11 bioclimatic variables. Pearson correlation coefficients (r) at the top right panels and scatterplots with LOESS smoothing lines at the bottom left panels with distributions of the variables in diagonal. The 11 bioclimatic variables are mean annual temperature (Tavg), difference between annual maximum and minimum temperature (TD), annual precipitation (Prec), wind speed (Wind), isothermality (BIO03), temperature seasonality (BIO04), maximum temperature of warmest month (BIO05), mean temperature of wettest quarter (BIO08), precipitation seasonality (BIO15), precipitation of coldest quarter (BIO19), and annual reference evapotranspiration (ET0).


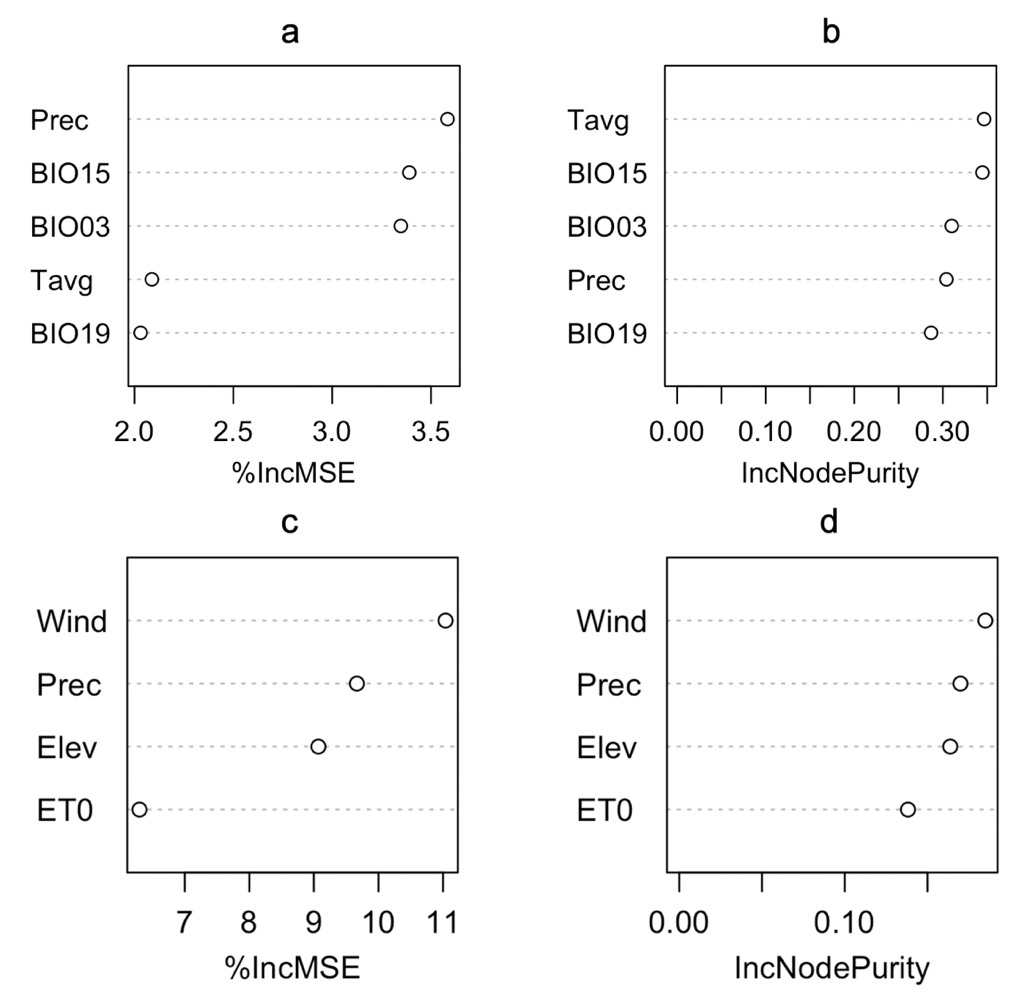


**Figure S2** Variable importance plots for the best random forest regression models for the two mosquitofish species. Mean annual temperature (Tavg), annual precipitation (Prec), isothermality (BIO03), precipitation seasonality (BIO15), and precipitation of coldest quarter (BIO19) are the predictors for the model for *G. holbrooki* (a and b). Elevation (Elev), annual precipitation (Prec), wind speed (Wind), and annual reference evapotranspiration (ET0) are the predictors for *G. affinis* (c and d). %IncMSE is the percentage of increase in mean square error if the value of this variable is randomly assigned (a and c). IncNodePurity is increase in node purity if the variable is added to the model (b and d).
